# Perioperative Intravascular Fluid Assessment and Monitoring: A Narrative Review of Established and Emerging Techniques

**DOI:** 10.1155/2011/231493

**Published:** 2011-07-12

**Authors:** Sumit Singh, Ware G. Kuschner, Geoffrey Lighthall

**Affiliations:** ^1^Department of Anesthesiology and Critical Care, Ronald Reagan UCLA Medical Center, David Geffen School of Medicine at UCLA, 757 Westwood Plaza, Suite 2231, Los Angeles, CA, 90095, USA; ^2^Division of Pulmonary and Critical Care Medicine, VA Palo Alto Health Care System, 3801 Miranda Avenue, Palo Alto, CA 94304, USA; ^3^Department of Anesthesia, Stanford University School of Medicine, 3801 Miranda Avenue, Palo Alto, CA, 94304, USA

## Abstract

Accurate assessments of intravascular fluid status are an essential part of perioperative care and necessary in the management of the hemodynamically unstable patient. Goal-directed fluid management can facilitate resuscitation of the hypovolemic patient, reduce the risk of fluid overload, reduce the risk of the injudicious use of vasopressors and inotropes, and improve clinical outcomes. In this paper, we discuss the strengths and limitations of a spectrum of noninvasive and invasive techniques for assessing and monitoring intravascular volume status and fluid responsiveness in the perioperative and critically ill patient.

## 1. Introduction

The ability to assess intravascular volume is an essential part of perioperative care and the management of perioperative hemodynamic instability. Insufficient intravascular volume can result in decreased oxygen delivery to tissues and organ dysfunction, while fluid overload states can contribute to the development of edema and organ dysfunction, including respiratory failure. The injudicious use of vasopressors and inotropes in the hypovolemic patient can be hazardous and increase the risk of a poor outcome.

Two concepts are relevant to assessments of fluid status in perioperative and critical care. *Euvolemia* describes a state of normal body fluid volume that allows adequate filling of the cardiac chambers and, in turn, makes it possible for the heart to produce a cardiac output that can meet the organism oxygen demand. In the setting of euvolemia, neither diuresis nor fluid administration is necessary. *Fluid responsiveness* describes the ability of the heart to respond to filling volume variations, modifying its stroke volume and consequently the cardiac output. From a patient management perspective, fluid responsiveness determines the extent to which circulatory homeostasis can be maintained with fluids alone versus the need for inotropes or vasopressors. An understanding of both concepts derives from the Frank-Starling relationship describing the changes in cardiac stroke volume in response to changes in cardiac preload. The ascending portion of the Frank-Starling curve will correspond to the fluid responsive phase of resuscitation, as seen with an increase in the cardiac output. Once the left ventricle reaches the plateau phase of the curve, fluid administration will not improve the cardiac output any further; it may lead to the adverse effects related to fluid overloading, such as hydrostatic pulmonary edema. 

In a broad sense, if euvolemia is the goal of fluid use in resuscitation, then fluid responsiveness reflects the process of working toward establishing euvolemia. In evaluating the various techniques for analyzing fluid status, it is helpful to contrast their utility in predicting fluid responsiveness versus euvolemia and to consider how these relative strengths and weaknesses may be paired with different clinical situations to yield accurate and meaningful information. Methods of interpreting intravascular volume range from clinical assessments such as inspection of veins and passive leg raising, to more invasive methods such as central venous and pulmonary artery catheterization, to newer technically intensive methods such as echocardiography and analysis of flow parameters.

## 2. Assessment of Fluid Status by Physical Examination

Only extremes of body water can be predicted by clinical examination. Tachycardia and hypotension and loss of skin turgor are associated with frank hypovolemia in unanaesthetized adults, while tenting of skin and sunken eyes and fontanels can be found in infants. The ability to detect hypervolemia is more dependent upon cardiac pathology where smaller grades of fluid excess can produce findings such as rales, a third heart sound, oxygen desaturation, jugular vein distension, and peripheral edema. In operative patients, experienced clinicians can often predict hypovolemia from the clinical situation. Fluid deficits resulting from a patient's *nil per os* (NPO) status and use of inhalational agents and positive pressure ventilation early in a case are usually indicative of relative hypovolemia, and anesthesiologists typically respond by increasing the volume of infused fluids. Blood loss and release of inflammatory mediators can also reduce blood volume, preload, and vascular tone in latter phases of an operation, when, again, additional boluses would likely be given with careful assessment of the blood pressure response. 

Depending on patient position and access, examination of neck veins and passive leg raising test can yield useful information. The *passive leg raising test* (PLR) delivers a reversible endogenous fluid challenge by increasing venous return resulting from elevating the legs to 45 degrees in a supine patient and evaluating its effect on blood pressure and heart rate. One simple described way to perform PLR is by using the pivotal motion of the bed to transfer a 45 degree recumbent patient into a horizontal position with supine trunk and elevated legs [1]. This technique recruits the splanchnic blood in addition to the blood from lower extremities, in contrast to simply elevating legs in a supine patient. PLR test has shown good correspondence with other derived indices to predict fluid responsiveness in patients with sepsis and pancreatitis [2] and has been specifically evaluated with transthoracic echo [3] and esophageal Doppler [4] in mechanically ventilated patients. Most operative situations will preclude the use of PLR and likely prompt the clinician to assess the response to a fluid bolus. However, in evaluating patients postoperatively, this simple technique may be useful.

## 3. Invasive Pressure Monitoring

### 3.1. Central Venous Pressure (CVP)

CVP is the measurement of pressures within the thorax in the superior vena cava (SVC) and serves as a reasonable surrogate for the corresponding right atrial pressures. CVP is the most widely used technique for measuring intravascular volume in critically ill patients [5]. 

**Figure 1:**
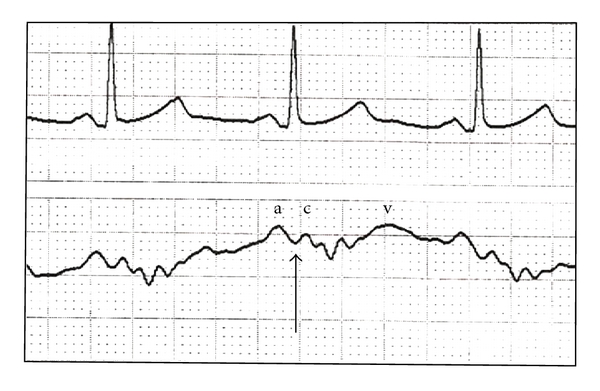
A typical CVP waveform (lower tracing) and accompanying electrocardiogram (upper). The a, c, and v waves are shown, along with the z point (arrow), indicating the appropriate time in the cardiac cycle for CVP measurement. All analyses need to occur at end expiration.

The CVP pressure tracing consists of three positive waves (a, c, and v) and two negative waves (x and y). The CVP is specifically measured at the “z” point of the CVP tracing which corresponds to the leading edge of the c wave (Figure 1). At this part of the cardiac cycle, the catheter tip is in continuity with the ventricle and hence affords the best estimate of cardiac preload [6]. As with the measurement of jugular venous distension, the reference point for measurement of the CVP is the midaxillary line in the fifth intercostals space. Numerical recordings of CVP are measured at end expiration, a time in the respiratory cycle where the opposing forces of lung elasticity and chest recoil are balanced and exert the least pressure on the central and pulmonary vasculature. In patients with forcible expiration, the true CVP may be better represented by a value at the start of expiration [6]. Failure to attend to these basic principles will lead to erroneous data with little reproducibility across multiple users and time points.

In our experience, single point estimates of CVP are of limited clinical value unless they are low (<5 mm Hg) and confirm an existing suspicion for hypovolemia. Trends and their correspondence to clinical evidence of organ function and perfusion help to create a more meaningful picture of fluid needs and euvolemia. The standard for testing volume responsiveness is to give a fluid challenge. This involves giving fluids to increase the CVP by 2 mm Hg and then determining whether it increased the CO [7]. In a study of 83 ICU patients, Magder and Bafaqeeh showed that patients who were able to increase the CVP by 2 mm Hg after receiving a bolus of approximately 500 mL of isotonic crystalloids over 10–30 minutes had a cardiac index increase of 300 mL/min/m^2^. The two important findings of the study were that only 4.5% of the patients with a CVP more than 10 mm Hg responded to a fluid challenge, also of the patients who had increase in CO, 42% had a simultaneous increase in blood pressure. Therefore, the two conclusions from the study were that patients with a CVP of more than 10 mm Hg were poor responders to volume infusion and that 10 mm Hg may represent euvolemia in most individuals. Second, blood pressure increase was not a good indicator of cardiac response to a fluid challenge [8].

The Society of Critical Care Medicine's Surviving Sepsis protocol recommends to fluid resuscitate a septic patient to a target CVP goal of 8–12 mm Hg and 12–15 mm Hg in a mechanically ventilated patient [9]. Though these recommendations are part of a sepsis care bundle that has been shown to improve survival, the CVP should not be interpreted in isolation, but rather in conjunction with the cardiac output. Depending on where the patient is on the Frank Starling curve, some patients may be adequately resuscitated at a CVP of 6-7 mm Hg, while others may still be hypovolemic at 10 mm Hg.

The discussion above applies to use of jugular and subclavian veins. If these sites are not available, femoral vein line pressures have been shown to be a good substitute for CVP. In various studies in spontaneously breathing and ventilated patients, inferior vena cava pressures have been shown to be around 0.5 mm Hg lower than the SVC pressures on average and rarely more than 3 mm Hg different [10]. Though the readings have been shown to be reliable in patients receiving mechanical ventilation and high positive end expiratory pressure, the same rule cannot be applied to patients with raised intra-abdominal pressures, as encountered during laparoscopy. 

A healthy person can have a CVP less than zero in upright position and still have an adequate CO and be euvolemic. Conversely, CVP can be high in a patient with poor ventricular function and low cardiac output or with a good ventricular function and volume overload [6]. As illustrated by these common scenarios, values derived from pressure readings are most useful when used in conjunction with a dynamic clinical response such as blood pressure or urine output, or some measure of cardiac output. Echocardiography, analysis of venous oxyhemoglobin saturation, and immediate changes in blood pressure have all been used in this regard. 

Another distinct advantage of central venous catheterization is the ability to obtain simultaneous measurements of pressure or pressure change and central venous saturations (ScvO_2_) with a single device. ScvO_2_ values of more than 70% are consistent with an adequate cardiac output and perfusion status, although this relationship is true only with adequate hemoglobin levels and stable oxygen requirements (VO_2_) of the body. The early goal-directed therapy for severe sepsis and septic shock has ScvO_2_ monitoring as an integral part of the resuscitation goals [11]. Intermittent blood sampling and catheters with special sensors are equally efficacious means of measuring ScvO_2_.

### 3.2. Pulmonary Artery Catheters (PACs) and Pulmonary Artery Occlusion (Wedge) Pressures

Aberrations in right heart compliance and pulmonary vascular resistance as may arise from cardiac and lung pathology can drastically alter the relationship between CVP and left heart preload. Pulmonary artery catheterization has therefore remained an attractive option to measure both right and left heart and pulmonary artery pressures in critically ill patients with such underlying pathology. The monitor is based on a balloon-tipped catheter that “floats” through the right atrium and ventricle and into the pulmonary artery as it is advanced. When “wedged” in one of the proximal branches of the pulmonary artery (PA), the catheter is in continuity with the left ventricle. With some minor gradients in between structures, as long as the catheter is the West Zone III of the lung and no mitral valve pathology is present, the following relationship exists.

### 3.3. Wedge Pressure *∝* Left Atrial Pressure *∝* Left Ventricular End-Diastolic Pressure (LVEDP)

When pulmonary vascular resistance is not elevated, the PA diastolic pressure approximates the pulmonary artery wedge pressure (PAWP) and provides a useful estimate of LVEDP without having to advance and wedge the catheter. 

Pulmonary artery catheters can also be used to measure mixed venous saturations and CO via the Fick principle by analysis of blood aspirated from the catheter's distal port. Typically, most CO measurements are made by electronic analysis of a thermal wash-out curve of fluid of a known temperature detected proximally at the point of injection and distally at a thermistor located 4 cm from the catheter tip. Continuous measurement of CO is accomplished with catheters containing an additional thermal filament and a computation module. The latter system requires occasional set-up time and calibration. Data from either of these catheters can be used to derive right ventricular ejection fraction (REF) and right ventricular end-diastolic volume (RVEDV). Thus, in addition to titration of fluids, the impact of inotropes, vasoconstrictors, and other interventions can be measured and followed. Low REF has been found to be helpful in identifying myocardial depression and as a marker of poor prognosis in septic patients [12]. In cardiac surgery patients, REF and RVEDV may be helpful in early detection of impaired right ventricular function secondary to right coronary artery stenosis [13].

As with the CVP, PAWP measurements are dependent on myocardial compliance. Multiple studies on ICU patients have shown that PAWP in acute illness correlates poorly or inconsistently with left ventricular end-diastolic volume (LVEDV) [14–17]. Comparisons of PAWP and right RVEDV index found the latter to correlate more reliably with changes in cardiac index (CI) [18, 19]. Though RVEDV by PAC has been found to be consistent with different methods of measurement, such as ventriculography [20] and angiography [21, 22], other investigators have found the data less convincing [23, 24]. De Simone et al. compared 3D echocardiography data with PAC-derived RVEDV and found no significant correlation between the two [25]. 

For most of the past three decades, use of the PAC was based on the uncontested assumption that targeted improvement of physiologic parameters to supranormal endpoints is a desirable strategy. This approach began in the early 1970s when Shoemaker and colleagues described a pattern of higher ventricular performance, oxygen delivery, and oxygen consumption which predicted survival in trauma patients [26]. Subsequent studies in surgical patients seemed to confirm the survival benefit of using the PAC to facilitate increasings CO and oxygen delivery [27, 28]. In high-risk surgical patients, a significant reduction in mortality (4% versus 30%) followed early implementation of a protocol attempting to reach supranormal cardiac indices (CI ≥ 4.5 L/min/m^2^), [29]). A similar reduction in mortality (5.7% versus 22%) was seen in high-risk surgical patients randomized to a protocol where an oxygen delivery index of greater than 600 mL/min/m^2^ was achieved by dopexamine infusion [30], and a later study of trauma patients demonstrated a decrease in mortality from 37% to 18% with a similar protocol of PAC-facilitated endpoints [31]. 

Using the PAC to facilitate increased oxygen delivery in medical and surgical ICU patients was the next natural step. However, therapy designed to achieve such supranormal values had no effect in one study where therapy was targeted to achieve a supranormal CI and normal SvO_2_ [32], did not improve outcome in patients randomized to receive an O_2_ delivery index of ≥600 mL/min/m^2^ in another [33], did not improve outcomes in septic patients where supranormal indices of DO_2_ and VO_2_ were attempted [34], and, finally, decreased survival in a study using the PAC to guide supranormal hemodynamics [35]. In the studies above, use of the PAC was intertwined with a specific protocol for achieving key therapeutic endpoints, thus seeming to negate the value of the technology, when the flaw may have been the endpoint the technology was used to achieve. A more recent generation of controlled studies comparing pulmonary artery and CVP catheters with less stringent, and in some cases clinician-generated endpoints, have again shown no benefit of the catheter *per se *in high-risk surgical patients [36], patients with shock and sepsis [37], and adult patients with the acute respiratory distress syndrome (ARDS) [38]. In the Canadian study of surgical patients, the PAC was associated with a higher incidence of nonfatal pulmonary embolism [36].

 Use of PACs has fallen over the last ten years due to the factors cited above, as well as due to higher complication rates [39], frequent misinterpretation of PAC data [40], and relative success with CVP-based methods for resuscitation in septic shock [11]. The American Society of Anesthesiologist Practice Guidelines recommend PAC for high-risk surgical patients only [41]. 

At present no study has demonstrated a positive association between PAC use for fluid management and survival [42]. Many of the randomized studies allowed physician exclusion of patients thought to benefit from the monitoring system, such as patients with heart failure and pulmonary hypertension where fluid management is only one of a handful of variables under active scrutiny and manipulation. Such use is likely to be highly individualized and unlikely to conform to a set protocol or study design that will allow a definitive statement of benefit. It is therefore prudent to be aware of the capabilities of the PAC and maintain an open mind about its potential value when a fuller physiologic picture may be necessary to make decisions regarding fluid responsiveness and volume status.

## 4. Cardiorespiratory Interactions and Dynamic Analysis of Fluid Status

Cardiac output and blood pressure interact with the respiratory system in a predictable manner according to the relationships indicated in Table 1. With positive pressure ventilation, venous return to the left ventricle is augmented, causing a rise in cardiac output and blood pressure during early inspiration. Later, the decrease in RV preload caused by positive intrathoracic pressure causes a drop in LV preload and systemic blood pressure [42]. Compared to the static hemodynamic parameters (CVP, PAWP), which are measured at one point in the cardiac cycle, dynamic parameters have been shown to be more accurate in assessment of intravascular volume and fluid responsiveness.

It is well recognized that pulsus paradoxus, or an inspiratory fall in systolic blood pressure by more than 10 mm Hg, is seen in critically ill patients with hypovolemia. With hypovolemia, the myocardium is at the steep portion of the Frank-Starling curve, so any minor variation in the preload with inspiration or expiration can cause appreciable changes in CO and blood pressure. Some rather direct and accurate inferences regarding intravascular volume can be made from analysis of arterial pressure waveforms and Doppler analysis of aortic blood flow. Ironically, while positive pressure ventilation is used, the accuracy of pressure-based measures of fluid responsiveness such as CVP is highly debated, while flow-based measurements achieve their highest accuracy. Indices of intravascular fluid and preload assessment derived from positive pressure ventilator-induced arterial blood pressure changes include systolic pressure variability, the respiratory systolic variation test, stroke volume variability, and respiratory changes in arterial pulse pressure.

### 4.1. Systolic Pressure Variability (SPV)

**Figure 2:**
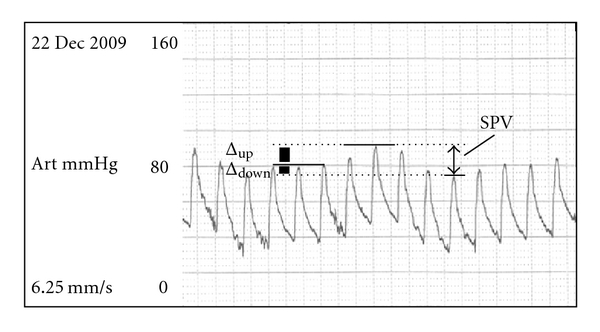
Arterial line tracings showing systolic pressure variation during the respiratory cycle. The pressure at end expiration defines baseline systolic pressure. SPV has two components, delta up and delta down, corresponding to systolic pressure waves reading at peak amplitude of early inspiration (the upward component or delta up) and at end inspiration (the downward component or delta down). The total amplitude variation or the sum of delta up and delta down is thus the SPV, shown with the arrow on the right of the diagram.

The range of blood pressure (difference between maximal and minimal systolic BP) during a single positive pressure breath is defined as SPV. The baseline systolic pressure is taken using a short apneic period. SPV has 2 components, delta up and delta down, corresponding to the difference between the baseline and the peak amplitude during early expiration and at end inspiration, respectively. SPV refers to the sum of delta up and delta down or the total amplitude variation (Figure 2).

Beside intravascular volume status, SPV and its two components can be affected by a multitude of factors which include arrhythmias, chest wall and lung compliance, abdominal pressure, method of ventilation (spontaneous or mechanical), and myocardial function [43–46]. All these factors remaining stable, variations in SPV will reflect changes in intravascular volume. With a normal myocardium functioning at the steep portion of the Frank Starling Curve, SPV is mainly due to delta down component, with a decrease in ventricular filling with positive pressure ventilation [44]. On the other hand, SPV is comprised of a delta up component in a patient with a failing myocardium, which is preload insensitive but responsive to afterload reduction with positive pressure ventilation [47]. The increase in pressure surrounding the heart decreases transmural pressure during systole (and hence afterload), thereby improving the ejection fraction. No exact values of SPV for determination of hypovolemia have been established. In a study by Rooke, SPV less than 5 mm Hg and delta down component less than 2 mm Hg showed absence of hypovolemia [48].

Other methods of analyzing fluid responsiveness from respiration-induced changes in arterial pulse waves have been described and validated. The r*espiratory systolic variation test* uses the slope obtained by the minimal systolic BP during a ventilator maneuver involving four successive incremental positive pressure breaths [49]. Greater negative slope was found to correlate with increasing amounts of hemorrhage [50]. *Respiratory change in arterial pulse pressure (DPp)* is defined as the difference in pulse pressure of the highest (Ppmax) and lowest magnitude (Ppmin) over several respiratory cycles, divided by the mean of the 2 values, expressed as a percentage. In one study, a DPp of 13% differentiated fluid responders from nonresponders [51]. *Stroke volume variability* is an index that is based on beat-to-beat variability in the arterial pulse during the respiratory cycle [52]. 

While all of these parameters can be measured manually via printouts from raw arterial pressure waves, a number of commercial systems discussed below provide automated analysis and trending along with means of measuring cardiac output. The PiCCO system (PULSION Medical Systems AG, Munich, Germany) derives volumetric parameters from arterial waveforms via a set of algorithms termed pulse contour analysis. The latter method, first described by Wesseling et al., computes the end-diastolic to end-systolic change in pressure over systemic vascular resistance (SVR) [53]. The beat-to-beat change in the shape of the arterial pressure waveform reflects the changes in the impedance of the aorta. Absolute values of aortic impedance and SVR obtained by thermodilution (using the same monitor) are necessary for calibration of the monitor; from this, values of stroke volume, cardiac output, and vascular resistance are derived. Thus, pulse contour is able to convert pressure-based signals such as pulse pressure and pulse pressure variability into analogous volume-based signals. Validation studies with the PAC showed that pulse contour analysis was able to track increases in stroke volume accurately in volume responsive postcardiac surgery patients [54, 55]. Likewise, through use of SVV as a gauge of volume responsiveness, investigators were able to predict intraoperative fluid requirements for obese patients undergoing bariatric surgery [56].

In principle, the monitor could indicate fluid responsiveness via pulse variability with mechanical ventilation as a stand-alone monitor, but, with calibration, an additional wealth of information is available. Unlike the PAC, the injectate signal is collected at one of the large arteries outside of the thorax (femoral, axillary, or brachial) and thus measures transpulmonary blood flow. Mathematical analysis of the transpulmonary dilution curve can be carried out further to derive actual fluid compartments within the thorax. Two of the derived parameters, extravascular lung water (EVLW) and global end-diastolic volume (GEDV), are of particular interest. Both GEDV and the related intrathoracic blood volume (ITBV) are volumetric preload parameters in contrast to the more commonly used pressure-derived parameters from CVP and PACs. Changes in GEDV have correlated better with changes in stroke volume than with changes in CVP [57]. The ability to measure extravascular lung water carries the hope of being able to differentiate and follow problems such as pulmonary edema and ARDS [58], the latter with a positive survival associated with which excess fluid use could be avoided [38, 59]. An observational study of septic patients by Martin demonstrated a better survival in those with lower EVLW [60]. Further, fluid therapy algorithms guided by GEDV and EVLW rather than clinical assessment can lead to faster resolution of pulmonary edema, shortened requirement for vasopressors, mechanical ventilation, and thereby ICU stays [61, 62]. 

An investigation of postoperative cardiac surgery patients showed that CO derived by PAC and transpulmonary method is clinically comparable [63]. The advantage of transpulmonary thermodilution over the PAC is that it does not require insertion into a pulmonary artery (reduce risk of arrhythmias and pulmonary artery rupture), though it does require a CVC and an arterial catheter and their attendant complications. Artifactual increases in CO introduced by tricuspid regurgitation in PAC use would not be present with use of transpulmonary blood flow measurement. Inability to measure mixed venous oxygen saturations can be partially compensated by trending central venous saturations via a CVC. 

Similar measurements using transpulmonary lithium dilution and detection by an ion selective electrode are the basis of another commercial system providing continuous CO and pulse wave analysis, the PulseCO (LiDCO Ltd, London, UK) [64]. The concentration-time curve for lithium ion calibration is read out on an attachment appended to the tubing on an existing artery after injecting from any intravenous line. The analytic algorithm is different from pulse contour in a few fundamental ways. The resulting “pulse power” computation is not sensitive to changes in pulse pressure amplitude found in distal arteries and is not affected by under- or over-dampening of arterial pressure transducers [65]. With these corrections built into the computational scheme, the LiDCO does not require a central arterial line. The less invasive nature of this system may also present advantages in certain patient populations such as those with limited central access or severe arterial disease. Despite these advantages, the LIDCO system does not give data such as GEDV and ITBV, and calibration measurements are limited by a desire to minimize lithium exposures (the number is, however, not defined by the manufacturer). The accuracy of the system is also low on patients on therapeutic lithium [65]. A more recent iteration of the LiDCO, the “LiDCO rapid,” uses a software-based nomogram to estimate stroke volume without the need for lithium calibration. Accuracy of the latter has been shown to differ from thermodilution-based cardiac output measurements in cardiac surgery patient; the thought being that the recalibration may be required in such dynamic situations where the arterial compliance fluctuates widely through the course of a case (i.e., pre- versus post-cardiopulmonary bypass) [66]. Pulse wave analysis systems including both LiDCO and PiCCO have been used in combination with passive leg raising to accurately predict fluid responsiveness [67]. 

Another recently introduced system called FloTrac/Vigileo (Edward Lifesciences, Irvine, Calif) has also been designed to function without external calibration. Instead individual demographic data (height, weight, age, and sex) are used with arterial waveform analysis to calculate CO, the principle being that SV is proportional to pulse pressure (PP). In addition, the FloTrac can also calculate SVV, and if a central venous catheter is present, SVR and central venous saturation can be obtained. Like the LiDCO rapid, the FloTrac features ease of use and requires only an arterial catheter [68]. A study comparing PiCCO with FloTrac found FloTrac/Vigileo (2nd generation software) to be as accurate as the PiCCO in predicting fluid responsiveness using SVV, although the threshold value of SVV for the FloTrac/Vigileo was lower (9.6%) than for the PiCCO (12.1%) [69]. A meta-analysis found FloTrac in acceptable agreement with CO derived with thermodilution techniques. However, the authors concluded that, in patients with rapid hemodynamic changes, hyperdynamic circulation, aortic regurgitation and intraaortic balloon pump counterpulsation, its use was questionable [68]. 

Pressure Recording Analytical Method (PRAM) (MostCare device, Vytech, Padu, Italy) is another device that estimates CO from the area under the arterial pressure wave. No external calibration is required as with FloTrac. A study in hemodynamically stable children comparing Doppler echocardiography found PRAM reliable, though more validation studies are required [70].

## 5. Echocardiography

From its initial use in outpatient settings in 1970s, echocardiography has found its place in all inpatient settings, ranging from the operating rooms to the intensive care unit (ICU). Different modalities of ultrasound include transthoracic echo being most noninvasive and portable, while the more invasive nature of esophageal Doppler and transesophageal echo is well tolerated in anesthetized patients. Nevertheless, all echographic techniques spare the patient the complications related to vascular access and indwelling devices. Also, in contrast to the PAC which is dependent on pressure measurements to make volume determinations, echocardiography relies on direct visualization of the cardiac anatomy and flow dynamics. Moreover, in patients where causes of circulatory failure overlap, echocardiography provides the ability to evaluate structural abnormalities (pericardial tamponade), contractility (ejection fraction), besides assessment of intravascular volume. 

### 5.1. Transthoracic Echo (TTE)

**Figure 3:**
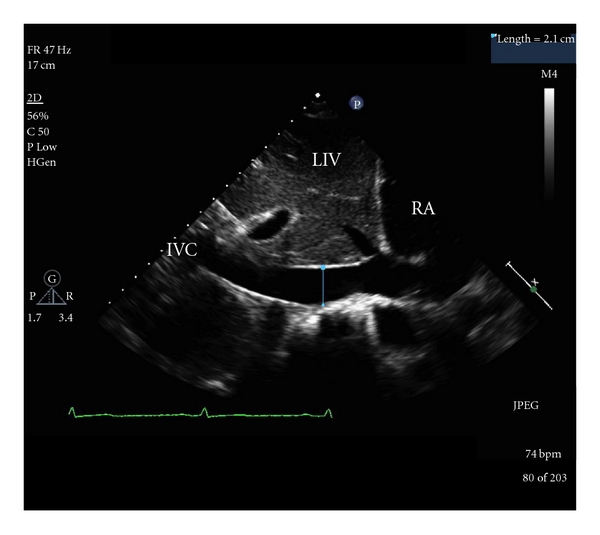
A subcostal view of the cavoatrial junction and adjacent structures. RA: right atrium; IVC: inferior vena cava; LIV: liver.

Improved image quality with portable study echo machines in the last decade has made TTE a popular tool for intravascular fluid assessment in the ICU. More recently, its use has been advocated in the perioperative settings due to its ability to provide quick, noninvasive functional and fluid assessment. Right heart preload can be reliably obtained by direct measurement of the inferior vena cava diameter (IVC) variations with respiration (Figure 3) and also by right and left ventricular end-diastolic volumes. One study showed that a 50% decrease in IVC diameter (caval index), seen by subcostal views with spontaneous breathing, correlated with an RA pressure of less than 10 mm Hg (mean SD 6 ± 5), as measured by CVP measurements [71]. Recent study in emergency department settings found caval index measurement a useful noninvasive tool for initial determination of CVP [72].

In mechanically ventilated patients, IVC variations with respiration (Delta DIVC) of 12% differentiated patients who responded with increased CO to a fluid bolus from nonresponders [73]. In another study with mechanically ventilated septic patients, the CVP and the IVC diameter increase on inspiration (distensibility index (dIVC)) was measured before and after a gelatin fluid challenge of 7 mL/kg. Response was measured as an increase in CI of 15% or more. dIVC greater than 18% predicted fluid responsiveness with a sensitivity and specificity of Ninety percent. CVP, on the other hand, correlated poorly with CI (*r* = 0.17, *P* = .45) and dIVC [74]. In spite of difficulty in visualizing IVC in postabdominal surgeries and obese patients, TTE provides a quick, noninvasive and reliable method of volume status of the patient. 

### 5.2. Transesophageal Echocardiography (TEE)

Besides cardiac anesthesia, TEE is now used routinely in other complex and long surgeries (e.g., liver transplant), especially among patients with known cardiac pathology. In our operating rooms, the quick availability of TEE machines makes this a first-line diagnostic modality for experienced users when unexpected hemodynamic instability is encountered. 

For general use, the main focus is on intravascular volume status assessment and overall cardiac function. In the ICU, TEE is recommended when diagnostic information cannot be adequately obtained by TTE [75]. Limitations of TEE include the lack of availability of trained personal and equipment, the requirement of adequate sedation or intubation, and the impracticality for continuous postoperative use. Also, TEE is an invasive procedure and contraindicated in patients with esophageal strictures and malformation.

As with TTE for IVC measurements to determine preload, TEE can be used to measure SVC collapsibility. Volume expansion was found to decrease SVC collapse, decrease RV stroke volume variation, and increase CI in a study in 22 mechanically ventilated ARDS patients with septic shock [76]. A collapsibility index of 36% was found to predict an 11% increase in CI following a fluid bolus in a separate study [77]. 

Both TTE and TEE allow visual estimation of ventricular volume. Ninety percent of the stroke volume is obtained by ventricular shortening in the short axis [78]. Therefore, measurement of the LV end-diastolic area using mid-transgastric short axis view by TEE can give a reliable estimate of LV end diastolic volume [79]. In practice, a qualitative assessment of LVEDA provides a quicker and quite reliable assessment, as Leung and Levine report that systolic cavity obliteration is 100% sensitive in detecting hypovolemia [80]. Fluid challenge in these cases can be followed by serial echo views of ventricular filling and correlation with the CVP. On the other hand, distended left ventricle with decreased EF would indicate minimal benefit from a fluid challenge. Additionally, a distended right ventricle and empty left ventricle may indicate right ventricular failure possibly from acute pulmonary embolism or pulmonary vascular disease. 

### 5.3. Esophageal Doppler Monitoring (EDM)

**Figure 4:**
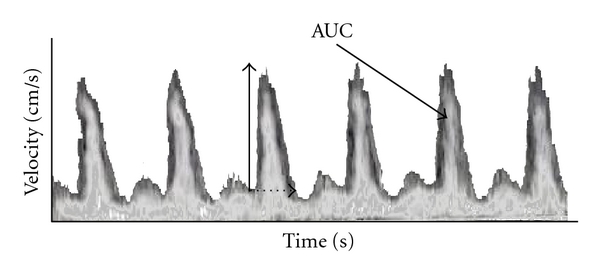
Esophageal Doppler tracings from descending aorta blood flow. Flow time and peak velocity are indicated by dashed and vertical arrows, respectively. Parameters derived from the latter measurements include corrected flow time (FTc), acceleration, and stroke distance. The area under the curve (AUC) is equivalent to stroke distance, the length traveled by an erythrocyte during a single cardiac cycle. Assuming that the flow is via a cylindrical path, the stroke volume (SV) is the product of aortic cross-sectional area and the stroke distance. Aortic blood flow is calculated from the product of heart rate and calculated SV and is not exactly equal to true cardiac output, as approximately 10% of total cardiac output is diverted to subclavian and cerebral arteries upstream to where flow is measured.

Using the Doppler principle to calculate blood flow velocity in the aorta, EDM can be used to provide minimally invasive continuous CO monitoring. EDM comprises of a small, thin, flexible probe with a Doppler transducer at the tip, inserted 35 cm into the esophagus, and has a tolerability similar to a nasogastric tube [81]. A continuous Doppler beam is used to generate flow velocity profile by analyzing reflected ultrasound waves from the moving red blood cells.  Figure 4 shows a flow-time tracing generated by EDM. Flow time is defined as the time needed by the left ventricle to eject the SV and is represented on the waveform from the start of the upstroke to its return to baseline. As flow time is also dependant on the heart rate, “corrected flow time” or FTc analogous to QTc in the electrocardiogram (ECG) is used in flow calculations. Area under the flow time curve is called the stroke distance. Combining with cross-sectional area can give the stroke volume. The cross-sectional area of the descending aorta can be obtained using nomograms (based on patients' height and weight) or measured directly with the M-mode by the EDM. Correction factor of 1.4 is used to adjust for blood lost to the coronaries and upper body circulation [81]. Additional analysis of flow velocity profile (peak velocity and upslope) can be used to evaluate left ventricular sensitivity to afterload and contractility. 

While nearly emerging devices for fluid management have sought to first establish themselves as similar to the “gold standard” PAC, the initial studies of EDM are relatively unique in their attempt to associate a positive clinical outcome with device use. In postsurgical patients, hypovolemia is a notorious cause of splanchnic hypoperfusion, prolonged hospital stays, and even increased mortality in moderate- to high-risk surgical patients [82–84]. With the use of a fluid management protocol built around using the continuous CO and FTc parameters of EDM monitoring, investigators were able to reduce the time to bowel motility, decrease the time to solid food intake, and shorten hospital stay [82, 85].

An analogous method for evaluation of cardiac output is based on estimation of blood flow via analysis of *electrical bioimpedance*. With electrical velocimetry (EV), all measurements are made via four ECG electrodes applied along one side of the patient's body. A small amplitude alternating current is applied to one pair of electrodes, while the other detects the change in electrical signal through the cardiac cycle. When blood moves forward through the ascending aorta during ventricular ejection, red cells tend to align more along the body's long axis, and create less impedance of electrical signals traveling along the same vector. Thus, from analysis of the electrical signal during the cardiac cycle, velocity and flow time parameters are generated and used to calculate stroke volume and cardiac output. 

Comparisons between EV and thermodilution cardiac output measurements show a good level of agreement over a wide range of outputs in adult cardiac surgery patients and critically ill adults [86, 87]. An excellent correlation (*r* = 0.97) was found between EV and cardiac output measurements made by the Fick method (using direct measurements of VO_2_) in children with congenital heart disease, and EV measurements correlated well with Doppler measurements made via transesophageal echo in cardiac surgery patients [88, 89].

Limitations to EDM use are similar to TEE. In addition, severe aortic regurgitation, severe lung disease, altered thoracic blood flow, and presence of intra-ortic balloon pump can alter accuracy of EDM [81]. 

## 6. Conclusion

Resuscitation of the hypotensive patient in the perioperative setting can be challenging. Accurate and timely assessment of fluid status and fluid responsiveness are cornerstones in the management of the hypotensive patient. Both hypovolemia and hypervolemia can be harmful. Goal-directed fluid management can result in the appropriate use of fluids, vasopressors, and inotropes, resulting in improved patient outcomes. Invasive, noninvasive, static, and dynamic monitoring methods have strengths and limitations. Continual monitoring of unstable patients through the resuscitation period utilizing a combination of techniques can improve the utility of hemodynamic information, improve the accuracy of assessments of volume status, and improve patient outcomes.

## Figures and Tables

**Table 1:** Cardiorespiratory interactions used to predict volume responsiveness are listed. RV: right ventricle; LV: left ventricle.

Mode of inspiration	RV preload	RV postload	LV preload	LV postload
Spontaneous	⇑	⇓	⇓	⇑
Controlled	⇓	⇑	⇑	⇓
